# Macular ganglion cell complex thickness after vitrectomy with the inverted flap technique for idiopathic macular holes

**DOI:** 10.5935/0004-2749.20220020

**Published:** 2025-08-21

**Authors:** Diego Valera-Cornejo, Marlon García-Roa, Paulina Ramírez-Neria, Veronica Romero-Morales, Renata García-Franco

**Affiliations:** 1 Retina Service, Mexican Institute of Ophthalmology, Querétaro, México; 2 National Autonomous University of Mexico, Mexico

**Keywords:** Retinal ganglion cells, Nerve fiber layer, Retina, Vitrectomy, Células ganglionares da retina, Camada de fibras nervosas, Retina, Vitrectomia

## Abstract

**Purpose:**

To analyze the macular ganglion cell-inner plexiform and retinal nerve fiber layer thicknesses after vitrectomy with the inverted flap technique for idiopathic macular holes.

**Methods:**

A prospective study was conducted on 28 eyes treated with surgery for idiopathic macular holes. The inverted internal limiting membrane flap technique assisted with Brilliant Blue staining (0.05%) was performed. Ophthalmologic examinations and quantitative analysis of the macular ganglion cell complex thickness were performed at baseline,1 and 3 months after surgery.

**Results:**

The preoperative mean thicknesses of the ganglion cell-inner plexiform layer and ganglion cell-inner plexiform layer + retinal nerve fiber layer were 88.9 and 124.8 µm, respectively. The mean ganglion cell-inner plexiform layer thicknesses at 1 and 3 months after surgery were reduced to 72.8 and 65.2 µm, respectively (p<0.001 and p<0.001, respectively). The mean postoperative ganglion cell-inner plexiform layer + retinal nerve fiber layer thickness was also reduced at 1 and 3 months (108.8 and 99.3 µm, respectively; p<0.001 and p<0.001, respectively). No significant difference was found between the preoperative and postoperative best-corrected visual acuities at 1 and 3 months (p<0.73 and p<0.14, respectively).

**Conclusion:**

The macular ganglion cell-inner plexiform layer and ganglion cell-inner plexiform layer + retinal nerve fiber layer thicknesses were significantly reduced after vitrectomy with the inverted flap technique assisted with Brilliant Blue staining (0.05%) for idiopathic macular holes.

## Abstract (pt)

**Objetivo:**

Analisar a camada plexiforme interna de células maculares e ganglionares e as espessuras da camada de fibras nervosas da retina após vitrectomia com técnica de *flap* invertido para orifício macular idiopático.

**Métodos:**

Estudo prospectivo de 28 olhos submetidos à cirurgia de orifícios maculares idiopáticos. Foi realizada a técnica de *flap* invertido da membrana limitante interna usando Azul Brilhante (0,05%). Os exames oftalmológicos e a análise quantitativa da espessura do complexo de células maculares ganglionares foram analisados na linha de base com 1 mês e 3 meses após a cirurgia.

**Resultados:**

A espessura média pré-operatória da camada plexiforme interna de células ganglionares e da camada plexiforme interna de células ganglionares ± camada de fibra nervosa da retina foi de 88,9 µm e 124,8 µm, respectivamente; a espessura média da camada plexiforme interna de células ganglionares com 1 mês e 3 meses após a cirurgia foi reduzida para 72,8 µm e 65,2 µm (p<0,001 e p<0,001, respectivamente). A espessura média da camada plexiforme interna de células ganglionares pós-operatórias + da camada de fibra nervosa da retina também foi reduzida com 1 e 3 meses (108,8 µm e 99,3 µm; p<0,001 e p<0,001, respectivamente). Nenhuma diferença significativa foi observada entre a acuidade visual melhor corrigida pré e pós-operatória com 1 e 3 meses (p<0, 73 e p<0, 14, respectivamente).

**Conclusão:**

A camada plexiforme interna de células maculares ganglionares e a camada plexiforme interna de células ganglionares ± as espessuras da camada de fibra nervosa da retina foram significativamente reduzidas após vitrectomia com técnica de *flap* invertido usando azul brilhante (0,05%) para orifício macular idiopático.

## INTRODUCTION

Idiopathic macular hole (IMH) is a full-thickness neurosensory retinal defect on the center of the fovea that mostly affects women (67%-91%) between the fifth and seventh decades of life and is bilateral in approximately 3%-27% of cases^(1)^. Vitrectomy associated with peeling of the internal limiting membrane (ILM) is the most widely used surgical procedure for the management of IMH owing to its higher success rates of 80-90%^(1,2)^. Triamcinolone acetonide (TA), indocyanine green (ICG), and Brilliant Blue G (BBG) are the vital dyes used to assist in the ILM peeling^(3,4)^. However, functional and histological damages have been reported after the procedure when assisted with ICG staining^(2,5,6)^, and some dyes such as BBG and TA appear to be safer for the retina^(7)^. Losses of nerve fibers and ganglion cells after ILM peeling have also been reported as a result of toxicity related to the staining substance, direct mechanical injury, or increased intraocular pressure during vitrectomy^(8)^. The peeling itself induces retinal changes on the inner surface of the retina, with minor changes on the thickness of the retinal nerve fiber layer (RNFL)^(9)^. Some controversy exists about the effect of ILM peeling and the use of vital dyes on the retinal ganglion cell complex (GCC)^(10-13)^. Peeling of the ILM may produce microscotomas and reduce retinal sensitivity^(14,15)^.

The innermost layers are the RNFL, ganglion cells (GC), and inner plexiform layer (IPL). These three layers correspond to the cell bodies, axons, and dendrites of GC, which in combination form the well-known ganglion cell complex. This complex serves as an early indicator of retinal damage due to the fact that these cells respond very early to ischemic changes^(16)^. However, not much research has been conducted regarding the effect of these dyes during the peeling of the ILM on the GCC, and different outcomes have been reported^(10,12)^. The recently available spectral-domain optical coherence tomography (SD-OCT) technology and algorithms provide better stratification and measurement of the GCC. The purpose of this report was to analyze the changes in the thickness of the macular GCC after macular hole surgery with the inverted flap technique.

## METHODS

### Study design

This study included a prospective case series of 28 eyes (28 patients) with IMH. All the eyes were treated with pars plana vitrectomy and ILM peeling with the inverted flap technique assisted with BBG staining. The Mexican Institute of Ophthalmology ethics committee approved the study. Patients from November 2018 through August 2019 were enrolled. Written informed consent was obtained from all the patients before enrollment in the study.

### Eligibility and exclusion criteria

We included patients (1) older than 18 years of age who had (2) an idiopathic full-thickness macular hole, defined as a full-thickness neurosensory retinal defect on the center of the fovea, detected on spectral-domain optical coherence tomography (SD-OCT; *SD-OCT Revo NX, Optopol Technology SA, Zawiercie, Poland*) and (3) no macular disease, including macular hole in the contralateral eye. Patients with a lamellar macular hole, an axial length >26 µm, a spherical equivalent exceeding -6 diopters, or a myopic macular hole were excluded. Those with histories of diabetic retinopathy, retinal neovascularization, trauma, inflammatory disease, vascular occlusion, or conditions (cataract or corneal opacities) that could interfere with the OCT measurement (signal strength <6) or visual acuity were also excluded.

### Subjects, follow-up, and optical coherence tomography analysis

The baseline examination included an evaluation of the best-corrected visual acuity (BCVA) using a Snellen chart (converted to logarithm of the minimal angle of resolution [logMAR] for analysis), slit-lamp evaluation, intraocular pressure measurement using a rebound tonometer (ICare, Tiolat AB, Helsinki, Finland), fundus examination, and tomographic analysis. The evaluations were performed at baseline, 1 and 3 months after the surgery. Previous pupil dilation with 0.8% tropicamide/5% phenylephrine (*T-P Ofteno, Sophia Laboratories, Guadalajara, Mexico*), the tomographic evaluation was performed using a three-dimensional protocol scan from the SD-OCT Revo NX. At baseline, the minimum diameter (minimal extent of the hole), base diameter (measured at the level of the RPE), height, hole form factor (HFF)^(17,18)^, macular hole index (MHI)^(19)^ and tractional hole index (THI)^(20)^ of the IMH were manually measured. The macular holes were classified according to the classification proposed by the International Vitreomacular Traction Study based on the minimum diameter as follows: large (>400 µm), medium (250-400 µm), or small (<250 µm)^(21)^. MHI was defined as the ratio of the hole height to the base diameter. THI was defined as the ratio of the maximal height to the minimum diameter, and HFF was calculated as the quotient of the sum of the right and left arm lengths divided by the basal diameter of the hole.

The GCC was measured using the ganglion cell analysis protocol, which is used to determine the boundaries of the RNFL and IPL and automatedly calculates the sum of the ganglion cell-inner plexiform layer (GCIPL) and/ or RNFL thicknesses. The segmentation software uses an automated algorithm to evaluate the GCC (GCIPL or RNFL + GCIPL) thickness. The complex is measured with two elliptical circles (the inner oval with horizontal and vertical diameters of 1.2 and 1.0 µm, respectively, and the outer oval with vertical and horizontal diameters of 4.0 and 4.8 µm, respectively) centered on the fovea. The mean and sectoral thicknesses (superotemporal, superonasal, superior inferonasal, inferotemporal, and inferior) were evaluated. The SD-OCT Revo NX ganglion cell analysis software does not allow a complete manual macular segmentation but allows relocation of the area to be analyzed. The SD-OCT ganglion cell analysis was performed at baseline and 1 and 3 months after vitrectomy. The macular closure type at 3 months was classified into two groups, type 1 (the hole closed without defect of the neurosensory retina at the fovea) and type 2 (the foveal defect persists but the rim of the hole is attached to the retinal pigment epithelium, and the cuff is flat)^(22)^.

### Outcome measures

The primary end point was the mean change from the baseline GCC (GCIPL and RNFL + GCIPL) thickness at 3 months. The secondary outcome measures included the mean change in the baseline BCVA logMAR score over time to 3 months, and the proportion and types of anatomical closure. A correlation analysis between the baseline anatomic parameters and visual acuity was performed. Associations between the type of macular hole closure and preoperative tomographic prognostic factors were also evaluated.

### Surgical procedure

A standard 25-gage 3-port pars plana vitrectomy was performed with the Constellation Vision System (Alcon, Fort Worth, Texas, US) in all the patients. Surgeries were performed by different vitreoretinal surgeons from the retina department of the institute. In eyes with cataract, the lens was removed by phacoemulsification, followed by intraocular lens implantation before vitreoretinal surgery. Core vitrectomy was performed, and the posterior vitreous was detached. Active suction with the probe was used to separate the posterior hyaloid; detachment of the posterior vitreous was not assisted with TA. With the infusion closed using a soft-tipped cannula attached to an insulin syringe, 0.1-ml BBG (*0.05% BBG, Aurolab, Madurai, India*) was injected in the vitreous cavity. Infusion was reopened after 30 seconds, and the dye was aspirated. The ILM was grasped at the temporal quadrant and peeled off with the ILM forceps (*Alcon Grieshaber-Switzerland/Alcon Labs, Inc., Fort Worth, TX*), covering an area of 2 disc diameters around the hole. Once peeled, it was not completely removed but was left attached at the edges of the hole, so small remnants of the ILM remain around it. Then, the ILM was manipulated over the hole from all sides until it became inverted (flap technique). Fluid/air exchange was performed (air pressure at 30 mmHg), and 20% sulfur hexafluoride (SF6) or 18% hexafluoroethane (C2F6) gas was used as a postoperative tamponade. The choice of gas was made according to surgeon preference. Sclerotomies were closed with a single 7-0 Vicryl suture if leakage was observed. Patients were asked to stay with a face-down position for at least 3 days (at least 16 hours a day). OCT examinations were performed after the gas bubble had completely dispersed from the vitreous cavity, which was 4 weeks for C2F6 and 2 weeks for SF6.

### Statistical analysis

Descriptive statistics were performed, reporting the variables with summary measures of central tendency and dispersion for quantitative variables and absolute and relative frequencies for qualitative variables. The normality of the quantitative variables was evaluated using the Shapiro-Wilk test, with a significance level of 0.05. Regarding inferential statistics, Student *T* tests and Pearson correlation were used, with a significance level of 0.05 using the Stata statistical package version 15.1 (*StataCorp. 2015, Stata Statistical Software: Release 15. College Station, Texas, US: StataCorp LP*).

## RESULTS

**Figure 1:**
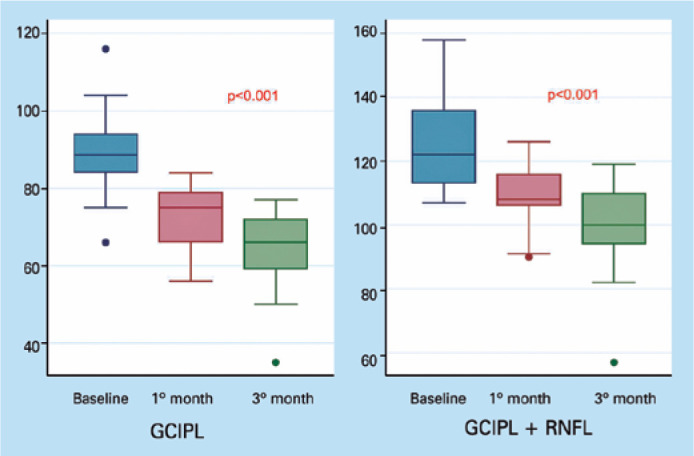
Ganglion cell complex thickness change at 1 and 3 months after surgery.

Twenty-eight eyes of 28 patients who underwent pars plana vitrectomy with BBG-assisted ILM peeling and the inverted flap technique for idiopathic macular holes in the retina service of the Mexican Institute of Ophthalmology were included. Twenty-four patients (85.7%) were female, with a mean age of 67.6 ± 5.2 years (57-79 years) and mean visual loss time of 10.4 ± 7.1 months (0-24 months). Baseline demographic and clinical characteristics are described in Table 1. Phacoemulsification with intraocular lens implantation combined with vitreoretinal surgery was performed in all the eyes. Twenty percent sulfur hexafluoride (SF6) was used as a postoperative tamponade in 93% of the eyes. The GCIPL thickness was significantly reduced from 88.9 ± 10.4 µm to 72.8 ± 8.3 µm after 1 month and to 65.2 ±10.4 µm at 3 months after surgery (p<0.001 and p<0.001, respectively). The GCIPL+ RNFL thickness was also significantly reduced from 124.8 ± 13.4 µm to 108.8 ± 9.9 µm after 1 month and to 99.3 ± 14.2 µm at 3 months after the procedure (p<0.001 and p<0.001, respectively; Figure 1).

**Table 1:** Baseline demographic and clinical characteristics (n=28)

Variable	Mean ± SD	Range
Age (years)	67.6 ± 5.2	(57-79)
Sex, n (%)		
Female	24 (85.7)	
Visual loss time (months)	10.4 ± 7.1	(0-24)
Macular hole stage, n (%)		
Small	0(0)	
Medium	2 (7.2)	
Large	26 (92.8)	
Minimum diameter (µm)	624.6 ± 162.2	(360-897)
Base diameter (µm)	1206.8 ± 217.1	(935-1847)
MH1	0.35 ± 0.07	(0.24-0.5)
HFF	0.59 ± 0.12	(0.4-0.83)
TH1	0.72 ± 0.29	(0.4-1.4)
Baseline BCVA, logMAR	1.19 ± 0.3	(0.65-1.6)
Baseline GC1PL thickness (µm)	88.9 ± 10.4	(66-116)
Baseline RNFL + GC1PL thickness (µm)	124.8 ± 13.4	(107-158)

SD= standard deviation; n= number; %= percentage; MHI= macular hole index; HFF= hole form factor; THI= tractional hole index; logMAR= logarithm of the minimum angle of resolution; GC= ganglion cell; IPL= inner plexiform layer; RNFL= retinal nerve fiber layer; BCVA= best-corrected visual acuity; mm= micrometer.

**Figure 2:**
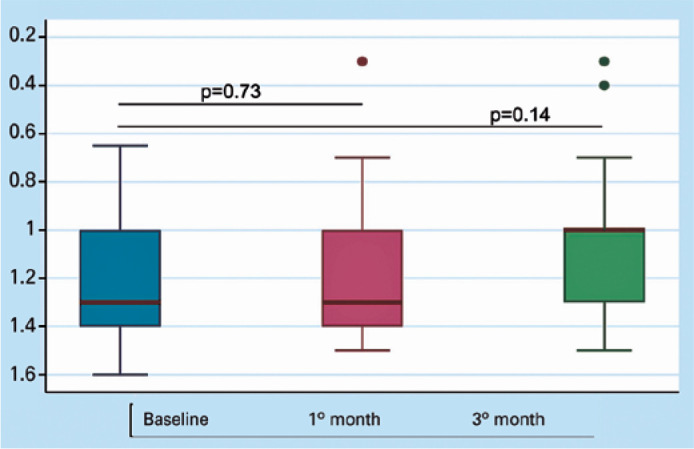
Best-corrected visual acuity change at 1 and 3 months after surgery.

The mean BCVA changed from 1.2 ± 0.27 logMAR units at baseline to 1.2 ± 0.30 at 1 month and 1.1 ± 0.33 logMAR units at 3 months. No statistically significant changes were observed at 1 month (p=0.73) or 3 months (p=0.14) after the surgical procedure (Figure 2). The primary anatomical success rate was 92%, as two eyes required a second surgery due to failure of macular hole closure. The final anatomical success rate was 100%. Of the different indexes and parameters, the HFF presented a statistically significant moderate correlation (p<0.05) with type 1 closure (Table 2).

**Table 2:** Correlations of the different indexes and parameters with visual acuity and type 1 closure at 3 months (n=28)

	Closure type 1		Visual acuity	
Variable	Coefficient	P value^*^	Coefficient	P value^*^
MHI	0.18	0.43	-0.30	0.17
HFF	0.48	0.03	-0.29	0.18
THI	0.27	0.22	-0.25	0.26
Minimum diameter	-0.35	0.11	0.42	0.05
Base diameter	-0.04	0.85	0.41	0.06

MHI= macular hole index; HFF= hole form factor; THI= tractional hole index.

* P value *by Pearson correlation*

## DISCUSSION

The inverted flap technique is considered effective and is used to enhance the anatomical closure rate of complicated cases (large or myopic macular holes), similar to those shown in our report, as almost all cases were large macular holes^(23)^. Whether anatomical and functional changes after ILM peeling for the management of macular holes are associated with the use of vital dyes or due to the micro traumas produced during ILM peeling is not well established.

We found that the reduction of the mean GCC (GCIPL and GCIPL + RNFL) thickness was evident 3 months after surgery, and several authors (described in Table 3) reported the same reduction in GCIPL and RNFL thicknesses in patients who underwent ILM peeling (assisted with Brilliant Blue staining) at 3 and 6 months^(10,11,13,24-27)^. However, Sevim et al. reported no significant reduction in the mean GCIPL thickness after BBG-assisted ILM peeling^(12)^. Others studies used ICG stain as a vital dye and reported a significant thinning of the RNFL^(28)^. Baba et al. compared the BBG and ICG stains and showed a significant reduction of the GCC at 6 months, but the retinal sensitivity and visual acuity were better in the patients in whom BBG staining was used during the vitrectomy^(29)^. Some studies found a significant thinning of the temporal retina only in the eyes with ILM peeling^(29,30)^. The mechanisms by which the GCC became progressively thinner after IMH surgery with ILM peeling are unclear. Our study did not determine whether the ILM peeling is directly responsible for the reduction of the thickness of the GCC because we did not include a non-ILM-peeling group. However, we believe that the direct mechanical damage to the ILM during the peeling rather than the cytotoxicity of the dye may have played a greater role in the GCC thinning postoperatively. The ILM is the basement membrane of the Müller cells, and the damage that occurs to these cells after ILM peeling has been electroretinographically and histologically demonstrated^(31,32)^. After the ILM peeling, retinal fragments can be observed on the retinal surface, regardless of the use BBG or ICG; this may lead us to believe that the mechanical traction from the pulling of the Müller cells during the peeling can be transmitted to the inner retina, damaging the entire region^(33)^. ILM peeling is also associated with microscotomas and reduced retinal sensitivity^(14,15,34)^. ILM peeling has been associated with dissociated optic nerve fiber layer (DONFL), which is seen in blue filter photography as a hatched retina with striae along the optic nerve fibers; these are dimples in the inner layers that are visible on OCT^(14,35,36)^. The idea of minimizing the iatrogenic trauma associated with ILM peeling can be achieved by decreasing the area to be peeled, which is reported by Michalewska et al. as a temporal ILM flap. With this technique, the risk of surgical trauma in the area of the papillomacular bundle is reduced, and unnecessary trauma to the RNFL is minimized; in addition, patients with a temporal ILM flap have a lower incidence of DONFL^(37)^. However, DONFL has not been consistently associated with reduced visual acuity or microperimetric changes, and DONFL may reflect the healing process of the weakened retinal structure after the peeling^(35,37)^. Multiple dark dots over the RNFL were observed when the appearance of the DONFL was examined using en face SD-OCT and named “concentric macular dark spots” (CMDS)^(38)^. Sabry et al. observed that after ILM peeling, two patterns of inner retinal layers changes occurred, CMDS with intact GCIPL and CMDS with localized defects in GCIPL; the latter was associated with a thinning of the GCIPL thickness at 6 months after surgery^(39)^. The authors suggested that even with a successful anatomical closure, inner retinal defects may play a role in the reduced postoperative visual acuity gain^(39)^.

**Table 3:** Reports evaluating the topographical changes of GCIPL thickness after macular hole surgery with ILM peeling

Author/year	Study type/ eyes	Vital dye/SD OCT	GCIPL reduction (*m*m)	Final GCIPL thickness (*m*m)	Final BCVA logMAR	Follow-up (months)
Present study 2019	PCS/28	0.05% BBG (Aurolab, Madurai, India)/Revo NX (Optopol Technology SA, Zawiercie, Poland)	- 23.6 ± 8.4	65.2 ±10.4	1.1 ± 0.33	3
Sevim et al. 2013^(12)^	PCI/70	0.25 % BBG (Fluoron GmbH, Ludwigsfeld, Germany)/	-0.81 ± 9.84	95.63 ± 7.46	0.39 ± 0.28	6
		RTVue-100 (Optovue, Fremont, CA, USA)				
Sabater et al. 2014^(11)^	RCS/ 32	0.25 % BBG (Fluoron GmbH, Ludwigsfeld, Germany)/Cirrus HD-OCT (Carl ZeissMeditec, Dublin, CA)	-5.46 ± 9.36	63.77	0.34 ± 0.32	6
Baba et al. 2014^¥(10)^	RCS/39	0.25 % BBG (ILM blue, DORC, Zuidland, The Netherlands)/ RTVue-100 (Optovue, Fremont, CA, USA)	--	40% of thinned areas^*^	0.20 ± 0.28	6
Hashimoto et al. 2015^(24)^	RCS/24	Not mentioned/RS-3000 (NIDEK, Gamagori, Japan)	-7± 8	67.2 ± 8.9	0.19 ± 0.34	6
Demirel et al. 2017^(13)^	RCS/18	0.025 % BBG + TB 0.15% TB (Dual membrane;DORC, Zuidland, The Netherlands)/Cirrus HD-OCT (Carl Zeiss Meditec)	--	52.61 ± 13.97	0.36 ±0.15	3
Baba et al. 2012^¥(29)^	RC/63	0.125% ICG	-11 ± 10	84.2 ± 10.8	0.37 ± 0.27	3-6
		0.25 % BBG (ILM blue; DORC, Zuidland, The Netherlands)/ RTVue-100 (Optovue, Fremont, CA, USA)	-11 ± 8	84.6 ± 8.4	0.25 ± 0.30	
Seo et al. 2015^(26)^	RC/58	0.25% ICG (Dianogreen Inj;Daiichi Pharmacy Co, Tokyo, Japan)/ Cirrus HD-OCT (Carl ZeissMeditec, Dublin, CA)	-11 ± 12.59	67	0.40	6

BBG= Brilliant Blue G; ICG= indocyanine green; TB= trypan blue; RCS= retrospective case series; PCS= prospective case series; RC= retrospective comparative study; SD-OCT= spectral-domain optical coherence tomography; GCIPL= ganglion cell-inner plexiform layer; BCVA= best-corrected visual acuity; logMAR= logarithm of the minimum angle of resolution; *m*m, microns.

¥The authors evaluated GCIPL +RNFL thickness.

* The authors evaluated the percentage of the total area of the GCC that was significantly thinner (red colored areas).

Brilliant Blue stain was used in all of our surgeries. Despite that some vital dyes have been reported to be toxic to retinal cells, we believe that the influence of the dye used is minimal. In addition, the BBG stain has been reported to have a protective effect on retinal cells and better anatomical and functional outcomes after macular hole surgery than ICG ^(29,40)^. ICG was reported to be toxic to the retinal GC in in vitro studies^(41)^ and could also remain in the vitreous cavity for 3 months after the procedure^(14,42)^. However, its toxicity during surgery is not clinically significant, and no clinical evidence supports its superiority over other dyes. Moreover, ICG was not used in our study^(43)^.

**Figure 3:**
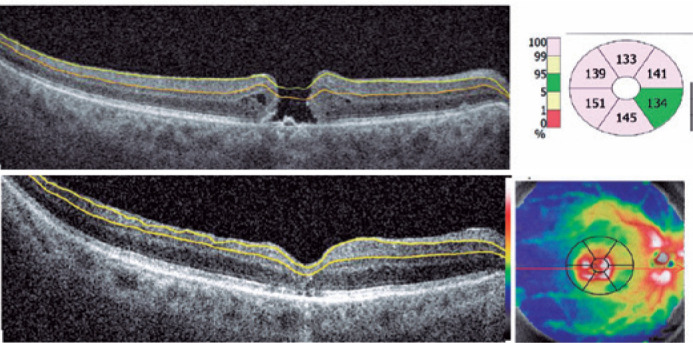
The ganglion cell analysis protocol determines the boundaries of the Retinal nerver fiber layer (RNFL) and inner plexiform layer (IPL), and automatedly calculates the sum of the ganglion cell-inner plexiform layer (GCIPL) and/or RNFL thicknesses. Left side: Boundary lines are drawn to measure the GCIPL thickness before and after macular hole surgery. Right side: The thickness (color-coded) map shows (clockwise) the thicknesses of the superior, superonasal, inferonasal, inferior, inferotemporal, and superotemporal sectors of the annulus.

The mean visual acuities at 1 and 3 months after surgery (logMAR 1.2 ± 0.30 and 1.1 ± 0.33, respectively) were unchanged and did not improve, which may be related to other factors rather than the GCC thinning because most eyes did not have good baseline predictive factors and had large macular holes, with long visual loss time and poor anatomical indexes (Table 1). The automatic segmentation performed by the ganglion analysis software of the SD-OCT Revo NX may be altered in eyes where the morphology is distorted by the macular hole (Figure 3). These measurement problems may be the reason for the higher baseline thickness reported and greater initial differences in the mean GCIPL and GCIPL + RNFL thicknesses in our study. A real manual segmentation may be useful for the measurement needed for these analyses.

Significant correlations between several OCT indexes (MHI, THI, and HFF) and postoperative BCVA have been reported in other studies^(19,20,44)^. Our study shows a trend similar to the aforementioned results, but it was not significant (Table 2). Contrary to our outcomes, those in other studies showed a strong correlation between MHI and postoperative BCVA as compared with other indexes^(19,44,45)^. Among several OCT indexes, we found a significant correlation between HFF (p=0.03) and closure type 1. However, other reports found that MHI strongly correlated with type 1 closure^(45)^. Determining the associations of several MH indexes with postoperative BCVA and closure type were not the primary objective of our study, and the small sample size could also explain this difference. The observation period was short (3 months), and the entire retinal thickness was reported to continue to diminish up to 24 months^(46)^. Even so, we report a significant reduction in GCC thickness in this short period, and longer follow-up periods are needed. Finally, the limitations of this study include its small sample size and design, the lack of a control group, and the short follow-up period.

In conclusion, the GCIPL and GCIPL + RNFL thicknesses are progressively decreased when the BBG-assisted ILM inverted flap technique is performed during vitrectomy for IMH treatment. Further research to understand the mechanisms related to the inner retinal damage during this procedure is necessary.
